# Circulating miR-146a predicts glucocorticoid response in thyroid eye disease

**DOI:** 10.1530/ETJ-23-0083

**Published:** 2023-09-22

**Authors:** Jacopo Manso, Simona Censi, Cristina Clausi, Ilaria Piva, Yi Hang Zhu, Alberto Mondin, Maria Chiara Pedron, Susi Barollo, Loris Bertazza, Giulia Midena, Raffaele Parrozzani, Caterina Mian

**Affiliations:** 1 Department of Medicine (DIMED), Endocrinology Unit, University of Padua, Padua, Italy; 2 Department of Women’s and Children’s Health, Pediatric Endocrinology Unit, Padua University Hospital, Padua, Italy; 3 Department of Molecular Medicine, University of Padua, Padua, Italy; 4 Department of Ophthalmology, University of Padua, Padua, Italy

**Keywords:** miR-146a, miR-21, thyroid eye disease, glucocorticoid sensitivity, glucocorticoid response, microRNA

## Abstract

**Objective:**

Thyroid eye disease (TED) is an immune-mediated disorder of the eye. Intravenous glucocorticoid (GC) is the first-line treatment for patients with active moderate-to-severe TED. However, the response rate is between 50% and 80%. There are still no simple and reliable markers of responsiveness to GC therapy. We aimed to explore the possible role of miR-146a and miR-21 as predictors of responsiveness to GC treatment in TED.

**Methods:**

We carried out a prospective longitudinal study on 30 consecutive adult patients with active moderate-to-severe TED and eligible for GC therapy. All patients received the standard GC treatment with methylprednisolone i.v. In cases of progressive worsening of Gorman Score for diplopia or with duction restriction <30° in at least two consecutive controls, patients also underwent orbital radiotherapy. Response to GC treatment was defined as a decrease of two or more points in the clinical activity score (CAS) or CAS <4/10 at 24 weeks. Circulating miRNAs were extracted from patients’ serum and quantified by real-time PCR.

**Results:**

Twenty-three (77%) patients responded to GC. Thyroid surgery, higher CAS, greater proptosis and higher pre-treatment circulating levels of miR-146a emerged as predictive factors of responsiveness to GC. A ROC analysis revealed that miR-146a could predict responsiveness to GC with a positive predictive value of 100%.

**Conclusion:**

This is the first study investigating the role of pre-treatment circulating miR-21 and miR-146a to predict responsiveness to GC in TED. miR-146a emerged as a simple, objective, new marker of GC sensitivity that could be used to avoid ineffective administration of GC therapy to TED patients.

## Introduction

Thyroid eye disease (TED) or Graves’ orbitopathy (GO) is an immune-mediated disorder of the eye in which cell-mediated response plays a crucial role and leads to oedema and deposition of glycosaminoglycans and collagens in the orbital fat and extraocular muscles (1). These changes cause enlarged extraocular muscles and orbital protrusion. TED is the major extrathyroidal manifestation of Graves’ disease (GD), but it may also be present in Hashimoto’s thyroiditis (HT) or rarely in euthyroid patients (euthyroid GO) (2).

The most common clinical manifestations of TED are proptosis, eyelid retraction, conjunctivitis, extraocular muscle hypertrophy with consequent reduction of ocular motility, diplopia and, in the most severe cases, optic nerve compression with consequent vision loss or corneal breakdown (3).

TED can significantly impact the patient’s appearance, vision and, most of all, quality of life (4).

The different concepts of disease ‘severity’ and ‘activity’ are fundamental in evaluating TED.

Simply put, disease severity reflects the type and extent of ocular involvement. Instead, disease activity refers to the typical biphasic course of the disease (Rundle’s curve) (5). TED presents in its natural course a progressive (active) phase lasting 6–18 months where inflammation is florid and a subsequent quiescent (inactive) phase where fibrotic processes are prevalent (4).

There are many grading systems for evaluating TED severity, such as the NO SPECS classification (6), the European Group on Graves’ Orbitopathy (EUGOGO) severity scale (7) and the VISA classification (8). The EUGOGO classification, the best grading system having been validated in clinical and research studies, grades TED into mild, moderate-to-severe and sight-threatening. The most recent EUGOGO guidelines confirm the clinical activity score (CAS), proposed by Mourits *et al.* (9), as the best-validated scoring system for evaluating disease activity (7). TED is defined as active if CAS is equal to or higher than 3 out of 7. A ten-item CAS is also approved to better evaluate disease activity during follow-up: in addition to the above 7 points, an increase in proptosis ≥2 mm, a decrease of eye movements in any direction of gaze ≥8° and a decrease of visual acuity ≥1 line on the Snellen chart during a period of 1–3 months are also considered. Active TED patients had a CAS greater than 4 of 10 points.

The disease-specific Graves’ ophthalmopathy quality-of-life questionnaire (GO-QoL) is a useful, validated method for evaluating the patient’s quality of life and improvement after treatment (10).

Medical treatments are usually reserved for active moderate-to-severe TED, as only in these cases have they proved to be efficacious with a good cost/benefit ratio.

Our patients were treated according to the previous EUGOGO guidelines (2016) since we started to recruit patients in 2018, when the current 2021 guidelines were not available yet. Therefore, intravenous glucocorticoid (GC) (methylprednisolone 4.5 g per cycle) is the standard first-line treatment used in our study, and we maintained the same treatment protocol throughout the whole study in order not to introduce bias (7, 11).

The rate of response to immunosuppressive treatment is between 50 and 80% according to published trials (7). For this reason, where first-line GC therapies fail, other second-line treatments are available, such as rituximab, tocilizumab or teprotumumab (only in the USA).

miRNAs are small, single-stranded, non-coding RNAs that play a role in regulating biological processes by inhibiting gene expression at the post-transcriptional level. They have emerged as crucial modulators of immunity and cellular processes (12).

Only a few studies have been conducted on miRNA expression in TED, and the results are sometimes conflicting (13, 14, 15, 16, 17, 18, 19). The vast majority of studies have investigated the expression of miR-21 and miR-146a in the retroorbital tissues or on circulating T-cells, but only one explored the expression of miR-146a in peripheral blood. Tong and colleagues found that miR-21 was upregulated in orbital fibroblasts from TED patients (*n* = 26) compared with healthy controls and acted as a mediator in TGF-β1-induced collagen production. Thus they showed that miR-21 promotes orbital muscle fibrosis in TED in *in vitro* models (15).

In two different studies, Jang *et al.* examined orbital fat and connective tissue samples from TED patients and showed that miR-146a could act as a negative regulator in the production of TGF-β-induced fibrotic markers and was locally overexpressed in TED patients compared with normal orbital adipose tissue (16, 20). In another study, Hu and collaborators collected and extracted active CD4+ T cells from the blood samples of six patients with active TED and six healthy subjects without TED. They found significantly lower levels of miR-146a in circulating CD4+T cells from TED patients, and that miR-146a could target NUMB in CD4+T cells to trigger ocular inflammation (18).

Given that about 40–50% of patients undergo a first treatment with glucocorticoids without benefit, the aim of this study was to investigate the possible role of serum miR-146a and miR-21 as predictors of responsiveness to GC treatment in TED patients.

## Materials and methods

### Patients

This prospective longitudinal study was carried out on 30 consecutive adult patients (age range 35–80) referred to the Endocrinology Unit of Padua University Hospital between 2018 and 2022 with previously untreated, active moderate-to-severe TED and eligible for GC therapy.

Inclusion criteria were as follows: (i) patients over 18 years of age with TED; (ii) active TED, defined as a CAS of three or more out of seven assessed according to EUGOGO guidelines; and (iii) moderate-to-severe TED, as defined by the latest EUGOGO guidelines (7). Exclusion criteria were as follows: (i) patients with previous GC treatment, contraindication to GC treatment (such as severe liver disease), hepatitis B or C, active infections, severe cardiovascular disease, psychiatric disorders, uncontrolled hypertension, uncontrolled diabetes mellitus, positive for anti-smooth muscle (ASMA), anti-mitochondrial (AMA), anti-liver–kidney microsomal (anti-LKM) antibodies, and pregnancy.

All patients taking part in the study gave written informed consent to the banking of their serum samples, with the approval of the Ethical Committee for Clinical Experimentation of Padua Hospital (Azienda Ospedaliera di Padova), protocol number: AOP1303. The study was also conducted in accordance with the Declaration of Helsinki.

### Clinical and ophthalmological assessments

Weight, height and body mass index (BMI) were obtained from all patients. The same specialist ophthalmologist team (R P and G M) determined CAS and measured ophthalmological parameters, such as proptosis (Hertel exophthalmometer), intraocular pressure, diplopia (Gorman Score), lid width and visual acuity of each patient. The patients’ quality of life was assessed using the EUGOGO disease-specific GO-QoL questionnaire (21).

All these parameters were measured at baseline, at the end of GC treatment and at 6 months from the last day of GC treatment.

### Treatment protocol and outcome definition

All patients received the standard GC treatment of 500 mg of methylprednisolone i.v. once weekly for 6 weeks, then 250 mg once weekly for 6 weeks (total 4.5 g) in accordance with the 2016 EUGOGO guidelines (11). In case of severe diplopia and ocular motility disruption during GC treatment patients also underwent orbital radiotherapy, which was given with a cumulative dose of 18 Gy per orbit fractionated in 12 daily doses over a 2-week period. In our study protocol, indications for orbital radiotherapy during GC treatment were progressive worsening of Gorman Score (from inconstant to constant, from intermittent to inconstant) or with duction restriction <30° in at least two consecutive controls.

Half the patients (*n* = 15, 50%) in our study underwent orbital radiotherapy in addition to the GC treatment.

All patients were treated with antithyroid drugs or levothyroxine, as appropriate, to maintain euthyroidism throughout the treatment and follow-up period.

The response to GC treatment was defined as a decrease in 2 or more CAS points or disease inactivation (CAS <4/10) at 24 weeks.

Blood samples were collected for research purposes before the start of GC treatment and on the last day of treatment.

### Laboratory tests

Electrochemiluminescence immunoassay (ECLIA) platforms were used to measure serum concentrations of thyroid-stimulating hormone (TSH), free thyroxine (FT4), free triiodothyronine (FT3) (Roche) and TSH-receptor autoantibodies (TRAb) (Maglumi®, Snibe Diagnostics, China).

The normal ranges, analytical sensitivities and intra- and inter-assay coefficients of variation were:

TSH: 0.27–4.2 mIU/L; 0.005 mIU/L; 3% and 8%

FT4: 12–22 pmol/L; 0.30 pmol/L; 2% and 5%

FT3: 3-6 pmol/L; 0.6 pmol/L; 3.5% and 3.6%

TRAB: <1.5 IU/L; <0.28 IU/L; 4% and 3%

### Measuring circulating miRNA by quantitative real-time polymerase chain reaction

Serum samples at baseline and on the last day of GC treatment were immediately prepared for miRNA quantification. RNA extractions from serum were performed with the DirectZol RNA Miniprep Plus Kit (cat. No. R2051, EuroClone, Milan, Italy) according to the manufacturer’s instructions. RNA was quantified by Nanodrop (Thermo Fisher Scientific). cDNA was synthesised with the TaqMan Advanced miRNA cDNA Synthesis Kit (cat. No. A28007, Thermo Fisher Scientific).

All real-time quantitative PCRs (qRT-PCR) were performed with TaqMan advanced miRNA assays (Thermo Fisher Scientific) for hsa-miR-146a-5p (assay ID 478399_mir) and hsa-miR-21-5p (assay ID 477975_mir) on the StepOnePlus real-time PCR system (Thermo Fisher Scientific). Expression levels were normalised to the hsa-miR-24-3p (assay ID 477992_mir) used as a housekeeping gene. All real-time reactions, including no-template controls, were run in triplicate. A pool of cDNA obtained from the serum of 19 healthy donors was used as the calibrator source. Data were analysed with the relative quantification (2^−ΔΔCt^) method, as described elsewhere (22, 23, 24).

### Statistical analysis

The statistical analysis was performed with the MedCalc (version 18.11.3) software. The normality of the distribution of all variables was assessed with the Shapiro–Wilk test. All data were expressed as means ± standard deviations (s.d.) for variables that were normally distributed and as medians with interquartile ranges (IQRs) for those that were not.

The power calculated from a Mann–Whitney *U* test expecting an effect size of 0.8 alpha = 0.05 (two tails) showed that a sample size of 27 was sufficient to detect the effect with Power = 0.8.

The Wilcoxon test was used to compare the baseline parameters with those at 24 weeks. The Mann–Whitney and *χ* ^2^ were used, as appropriate, to compare the clinical, biochemical and molecular data of patients who responded to GC treatment ± orbital radiotherapy with those who did not. Rank correlations between miRNA and the clinical and biochemical parameters were calculated by Spearman’s rho, where appropriate.

Concerning the multivariate analysis, we fit two regression models: one logistic regression model with treatment response and the baseline clinical, biochemical and molecular parameters as predictors; one multiple linear regression with miR-146a and the same baseline clinical, biochemical and molecular parameters as predictors.

A receiver operating characteristic (ROC) curve analysis was performed to identify a miR-146a expression level cut‐off at baseline able to predict which patients would respond to the GC treatment.

All results were considered statistically significant at *P* < 0.05.

## Results

Table 1 shows the patients’ clinical, biochemical and ophthalmological characteristics at baseline and 24 weeks after GC treatment ± orbital radiotherapy. After the treatment, we observed a statistically significant reduction in CAS and TRAb, improvements in visual and appearance QoL and diplopia, and no significant changes in BMI and proptosis in the entire TED population. At the end of the study, 23 (77%) patients were considered responders to GC treatment. Ten patients (33%) developed at least one side effect related to GC therapy, the majority of which were minor, such as gastric symptoms not improving with proton pump inhibitors. However, we also observed two liver toxicities, one development of major depression, one severe infection requiring hospitalisation (pneumonia), and in one case the onset of diabetes mellitus requiring therapy. Major side effects were managed by suspending GC until resolution of the acute phase and subsequent resumption of treatment.

**Table 1:** Characteristics of patients participating in the study at baseline and at 24 weeks after treatment.

Characteristic	*N* = 30		*P*-value
	Before treatment	At 24 weeks after treatment	
Age (median (IQR))	56 years old (46–62) (95% CI 55–58)		
Sex			
M	12 (40%)		
F	18 (60%)		
Body mass index (median (IQR))	24 (23–29) (95% CI 24–27)	24 (22–26.8) (95% CI 23–26)	0.08
Duration of thyroid eye disease (median (IQR))	2 months (1–7) (95% CI 1–3)		
History of thyroid disease			
Graves’ hyperthyroidism	28 (93%)		
Hashimoto’s thyroiditis	1 (3%)		
Euthyroid TED	1 (3%)		
Previous RAI			
Yes	4 (13%)		
No	26 (87%)		
Clinical activity score (median (IQR))	4 (3-5) (95% CI 3–5)	2 (1-4) (95% CI 1–2)	**<0.0001**
TRAb (median (IQR))	8.6 IU/L (6.8–12.3) (95% CI 7.5–12.2)	5.7 IU/L (2.8–9.8) (95% CI 3.9–7.3)	**0.0075**
Proptosis measurement (median (IQR))	19.5 mm (17–22) (95% CI 19–20)	19.5 mm (17–21) (95% CI 19–21)	0.8
GO-QoL visual function (median (IQR))	58 (43–78) (95% CI 56–63)	66 (44–88) (95% CI 57–81)	**0.02**
GO-QoL appearance (median (IQR))	69 (50–81) (95% CI 56–69)	81 (50–88) (95% CI 69–81)	**0.01**
Diplopia (Gorman score)			**0.0378**
Absent	4 (13%)	7 (23%)	
Intermittent	5 (17%)	6 (20%)	
Inconstant	10 (33%)	8 (27%)	
Constant	11 (37%)	9 (30%)	
At least one side effect		10 (33%)	
Responders to treatment (%)			
Yes		23 (77%)	
No		7 (23%)	

GO-QoL, Graves’ ophthalmopathy quality-of-life questionnaire; TRAb, TSH receptor antibodies. Bold indicates statistical significance.

**Figure 1:**
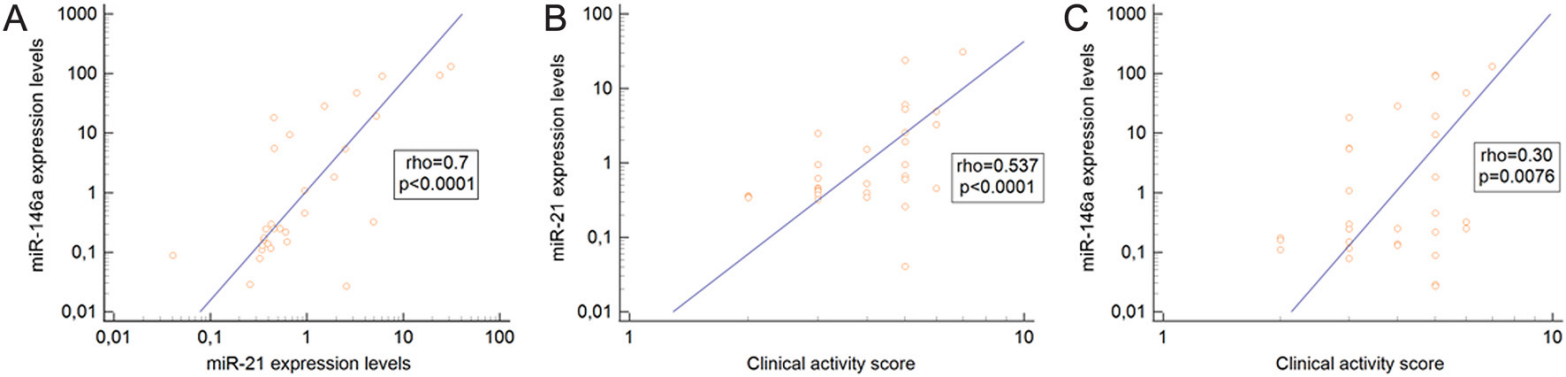
(A, B, C) Correlations among baseline serum miR-146a, miR-21 and clinical activity scores in TED patients.

The baseline circulating miR-146a and miR-21 expression levels were positively correlated with each other (*P* < 0.0001) (Fig. 1A). Moreover, both miR-146a and miR-21 were positively correlated with CAS (*P* = 0.0076 and *P* < 0.0001, respectively) in TED patients (Fig. 1B and C).

Moreover, gender, age, smoking habits and duration of TED did not influence miR-146a or miR-21 expression (see Supplementary Materials, see section on supplementary materials given at the end of this article).

We ran also a multiple linear regression with miR-146a as a dependent variable and the baseline clinical, biochemical, and molecular parameters as predictors: only baseline CAS (coefficient 10.17 *P* = 0.0022) and pre-treatment miR-21 (coefficient 2.83 *P* = 0.0005) emerged as independent predictors of pre-treatment miR-146 expression levels.

### Risk factors associated with glucocorticoid response

Table 2 presents a comparison of the baseline characteristics of TED patients responsive to GC treatment with those who were unresponsive. Thyroid surgery after the onset of TED, higher CAS, greater proptosis and higher circulating levels of miR-146a emerged as predictive factors of response to GC treatment.

**Table 2:** Univariate analysis of predictor factors for response to glucocorticoid therapy in patients with active moderate-to-severe thyroid eye disease.

	Non-responsive *n* = 7	Responsive *n* = 23	*P*-value
Age (median (IQR))	55 years old (37–62) (95% CI 37–62)	58 years old (54–58) (95% CI 55–58)	0.34
Sex			0.32
Male (%)	2 (29)	10 (44)	
Female (%)	5 (71)	13 (56)	
Body mass index (median (IQR))	25 (24–28) (95% CI 22–29)	25 (23–30) (95% CI 23–28)	0.80
Smoking habits			0.12
Yes (%)	5 (71)	11 (48)	
No (%)	2 (29)	12 (52)	
Thyroid surgery			**0.0018**
No (%)	5 (71)	5 (22)	
Before thyroid eye disease onset (%)	0	5 (22)	
After thyroid eye disease onset (%)	2 (29)	13 (56)	
Duration of thyroid eye disease (median (IQR))	2 months (1–9) (95% CI 1–10)	2 months (1–7) (95% CI 1–3)	0.91
Orbital radiotherapy			0.54
Yes (%)	3 (43)	12 (52)	
No (%)	4 (57)	11 (48)	
Clinical activity score before treatment (median (IQR))	3 (3–4) (95% CI 2–4)	5 (3–5) (95% CI 4–5)	**0.0059**
Diplopia before treatment*			0.57
Absent (%)	1 (13)	3 (13)	
Intermittent (%)	2 (29)	3 (13)	
Inconstant (%)	2 (29)	8 (35)	
Constant (%)	2 (29)	9 (39)	
Proptosis (median (IQR))	18 mm (17–19) (95% CI 17–19)	20 mm (18–23) (95% CI 19–21)	**0.018**
TRAb level (median (IQR))	15.9 IU/L (4.8–31.7) (95% CI 4.8–31.7)	8.1 IU/L (6.8–14.2) (95% CI 7.1–9.9)	0.17
GO-QoL visual function (median (IQR))	56 (50–75) (95% CI 50–75)	57 (43–78) (95% CI 56–63)	0.8
GO-QoL appearance (median (IQR))	50 (25–69) (95% CI 25–69)	69 (56–81) (95% CI 63–75)	0.09
miR-146a expression levels (median (IQR))	0.18 (0.15–0.25) (95% CI 0.15–0.25)	1.22 (0.17–22.82) (95% CI 0.29–7.2)	**0.01**
miR-21 expression levels (median (IQR))	0.27 (0.08–0.42) (95% CI 0.08–0.43)	0.46 (0.12–1.53) (95% CI 0.32–0.69)	0.07

^*^According to Gorman Score: 0 = absent; 1 = intermittent; 2 = inconstant; 3 = constant. GO-QoL, Graves’ ophthalmopathy quality-of-life questionnaire; TRAb, TSH receptor antibodies. Bold indicates statistical significance.

On multivariate logistic regression analysis, only baseline CAS (OR 12.00, 95% CI 1.15–125.23) and baseline proptosis (OR 2.78, 95% CI 1.03–7.4) predicted GC treatment response.

However, given the importance of obtaining a simple, objective marker potentially able to predict response to GC treatment from the outset in order to avoid administering ineffective therapy to patients, we then focused on the baseline circulating miRNA.

**Figure 2:**
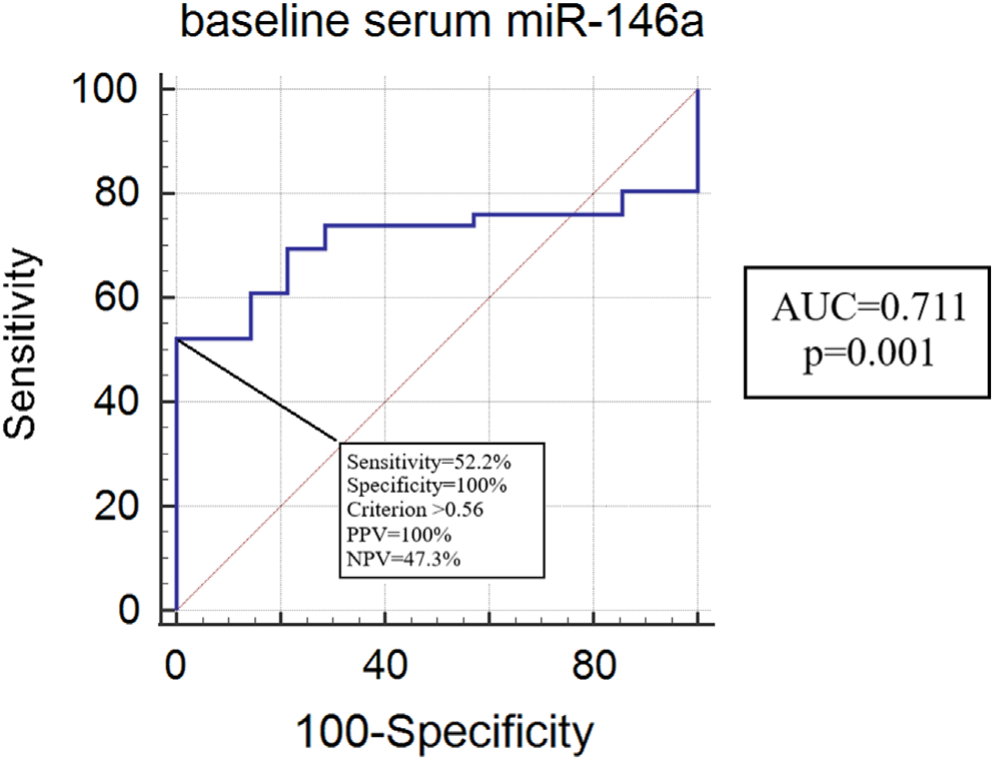
Receiver operating characteristic (ROC) curve analysis of serum miR-146a at baseline and responsiveness to GC treatment in thyroid eye disease patients. NPV, negative predictive value; PPV, positive predictive value.

ROC analysis revealed that with a cut‐off of 0.56 for miR-146a we could predict responsiveness to GC with a sensitivity of 52.2%, a specificity of 100%, a positive predictive value (PPV) of 100% and a negative predictive value of 47.3% (*P* = 0.001, area under the curve 0.711) (Fig. 2).

## Discussion

TED represents a very challenging condition for endocrinologists as it has a high impact on quality of life, frequently impairing usual activities of daily living and having a profound effect on self-image perception in moderate-to-severe cases (25). Despite the multidisciplinary approach to diagnosis and therapy routinely pursued, around 23–65% of TED patients do not respond adequately to GC therapy at the medium dosage (4.5 g) (26, 27, 28, 29). Furthermore, a careful review of the literature by Zang and colleagues revealed that the rates of cardiovascular and hepatic morbidities and mortality associated with iv GC therapy are not negligible – around 6.5% and 0.6%, respectively (30). For all these reasons, a simple, reliable biomarker able to predict response to GC therapy would be extremely helpful.

In our study, we had a GC response rate of 77%, in line with other published studies (7) and 33% of our TED patients experienced side effects, similar to the incidences reported in other clinical trials (20–56%) (30, 31).

miR-21 and miR-146a are considered circulating ‘inflammamiRNAs’, that is to say, miRNAs that control the inflammatory pathways regulating NF-κB and NLRP3 (32, 33). miR-21 is induced by pro-inflammatory cytokines (34) and regulates inflammatory processes by context-specific promotion or inhibition of NF-κB/NLRP3 pathways (33). In TED, miR-21 acting as a TGF-β1 modulator seems to trigger orbital muscle fibrosis (15). Similarly, miR-146a is induced in pro-inflammatory conditions, such as in response to lipopolysaccharide, IL-1β and TNF-α (35). Moreover, knockout mice for miR-146a exhibited hyper-reactive and pro-inflammatory circulating neutrophils (36). Besides, there is some evidence that miR-146a participates in the pathogenesis of several autoimmune diseases, such as rheumatoid arthritis, multiple sclerosis and systemic lupus erythematosus (37, 38, 39). Thus, overall miR-21 and miR-146a appear to act as negative regulators of inflammation, induced by inflammation itself.

With respect to TED, only Wei *et al.* have investigated circulating miR-146a in plasma from 14 active TED patients. They found that plasma miR-146a was under-expressed in active and inactive TED patients compared with the control group (19). In contrast to our findings, they also observed a negative correlation with the clinical activity of TED evaluated with CAS. The discrepancy may have to do with the different populations examined: we selected only patients with active moderate-to-severe TED as candidates for GC therapy. In our series, both miR-146a and miR-21 were positively correlated with CAS, which is better explained given that both miRNAs increased in response to inflammatory status, which deteriorates as CAS increases. Interestingly, serum miR-21 and miR-146a were positively correlated in TED patients, confirming a possible common pathophysiological mechanism linked to inflammation and the development of fibrosis in TED. Interestingly, IGF1-R is a recognised target gene of miR-146 and its role in the development of TED is well established (17, 40).

Of all the predictors of responsiveness to GC therapy that we found, only baseline circulating miR-146a is particularly promising, as it is objective and quantifiable, unlike CAS and proptosis, which are partially operator-dependent parameters. Only one other study, by Shen and colleagues, has analysed circulating miRNAs as a possible predictor of GC sensitivity in TED (*n* = 35). They found that patients resistant to GC had lower circulating miR-224-5p, but it was only in combination with TRAb levels that they reached a PPV of 91.37% for GC response pre-treatment.

In our sample, circulating miR-146a was higher in GC-responsive patients, and with an appropriate cut-off (above 0.56), it had a PPV of 100%. If confirmed by further studies, our findings may guide the pharmacological management of TED patients, so that ineffective and even potentially harmful choices may be avoided. TED patients with pre-treatment serum miR-146a above the cut-off should undergo i.v. GC therapy, while those with lower values could immediately be candidates for second-line therapies, thereby avoiding wasting time with GC therapy and potential related side effects. We can speculate that overexpression of circulating miR-21 and miR-146a signals an increased inflammation status in TED and therefore a clinical setting where there is a greater likelihood of response to particular immunosuppressive therapies.

While waiting for the discovery of robust predictive markers of GC response, it would be advisable to consider the possibility of an early treatment stop in non-responders patients, especially if at risk of GC side effects, since most TED patients respond as early as 6–8 weeks (41).

We are aware that our study has some significant drawbacks. The present series is quite limited, but the study is prospective and consecutive, and we enrolled more patients than the majority of previous studies focusing on miRNAs in a TED scenario. Another limitation is the definition of response to treatment used in our study: we used the improvement of the CAS alone, but a better evaluation is provided by the recent Composite Index Score proposed by EUGOGO which should be used to confirm our results in future studies.

Furthermore, the heterogeneity of our treatment protocol, with half the patients who underwent i.v. GC also undergoing orbital radiotherapy, should be highlighted. Indeed, orbital radiotherapy is considered a second-line treatment in combination with i.v. GC, particularly if diplopia and/or restriction of extraocular motility are present. However, orbital radiotherapy could also be used as a first-line treatment in combination with i.v. corticosteroid when significant involvement of the retroorbital muscles is present determining progressive diplopia, although randomised clinical trials supporting this possibility are still lacking (42, 43).

Further studies on a larger scale are needed to confirm our findings and develop a desirable composite multi-parameters algorithm to have the highest chances to correctly predict the response to GC treatment, including miRNAs as well as other parameters like proteomics, clinical and hormonal data.

## Conclusion

This is the first study investigating the role of pre-treatment circulating miR-21 and miR-146a in predicting responsiveness to GC therapy in patients with active moderate-to-severe TED. Serum pre-treatment miR-146a emerged as a reliable, simple and objective new marker of GC sensitivity. Using an appropriate cut-off, we could avoid unnecessary treatment with GC and instead direct the therapeutic strategy *ab initio* towards a second-line treatment, especially in an era of precision medicine. Of course, further studies are needed to verify our findings and make them applicable in daily clinical practice.

## Supplementary Materials

Supplementary Figure 1. Association between pre-treatment serum miR-146a expression levels and gender. Ns= not significant

Supplementary Figure 2. Association between pre-treatment serum miR-146a expression levels and smoking habits. Ns= not significant

Supplementary Figure 3. Correlation between pre-treatment serum miR-146a expression levels and patient’s age at diagnosis. Ns= not significant

Supplementary Figure 4. Correlation between pre-treatment serum miR-146a expression levels and duration of thyroid eye disease. Ns= not significant

## Declaration of interest

The authors declare that the research was conducted in the absence of any commercial or financial relationships that could be construed as a potential conflict of interest.

## Funding

This research did not receive any specific grant from any funding agency in the public, commercial or not-for-profit sector.

## Author contribution statement

JM contributed to the conception and design of the study. JM, IP, SC, CC, YHZ, AM, MCP, SB, LB, GM, RP and CM compiled the database. JM performed the statistical analysis. JM and IP wrote the first draft of the manuscript. JM, IP and CM wrote sections of the manuscript. All authors contributed to revising the manuscript and read and approved the submitted version.
