# Supplementary figures and images for: Circulating miR-146a predicts glucocorticoid response in thyroid eye disease
Source: Eur Thyroid J. 2023 Sep 22;12(5):e230083. doi: 10.1530/ETJ-23-0083 (PMC10563606; doi:10.1530/ETJ-23-0083)

miR-146a expression levels

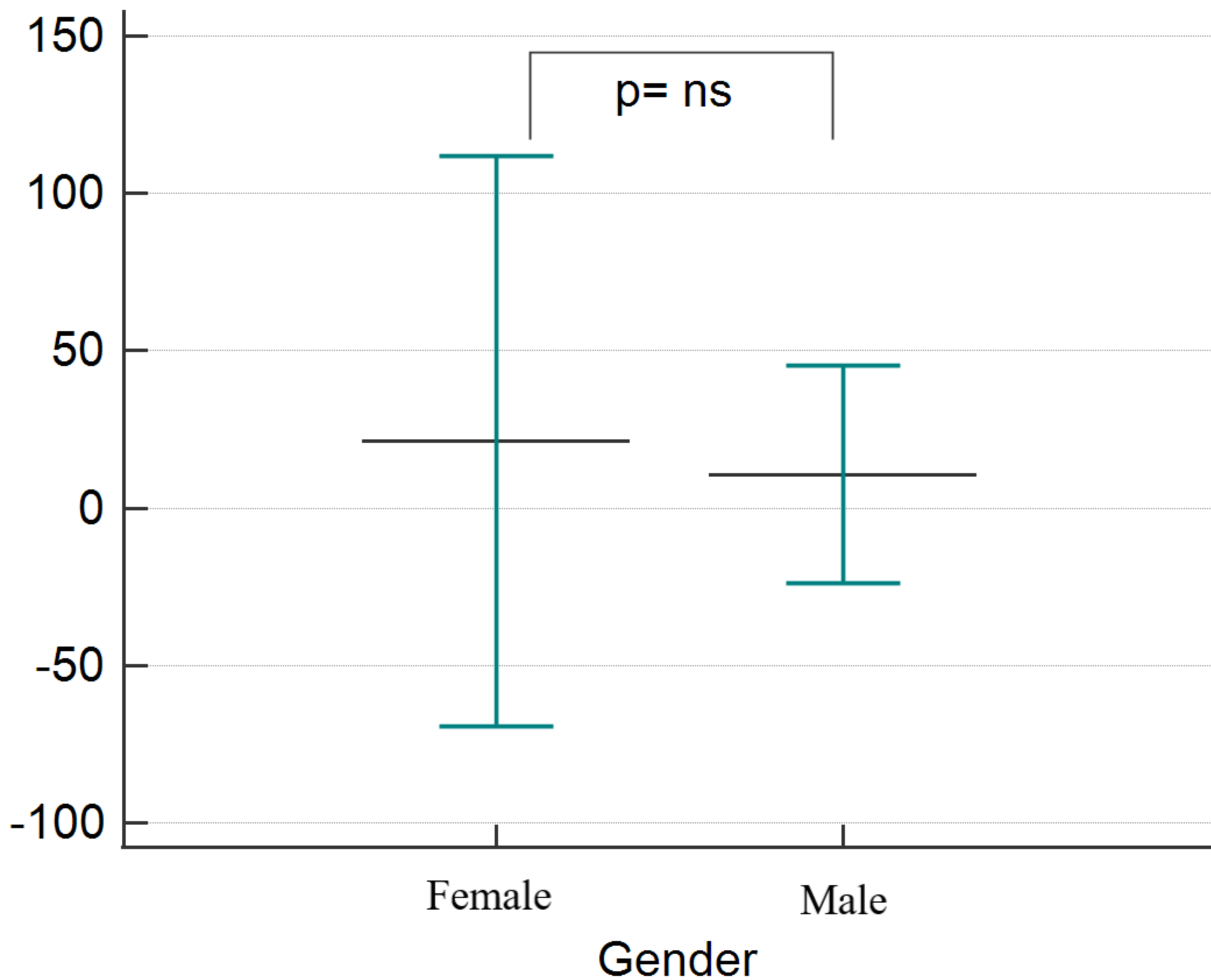

Supplement: Supplementary Figure 1. Association between pre-treatment serum miR-146a expression levels and gender. Ns= not significant [file supplementary_figure_1.pdf]

miR-146a expression levels

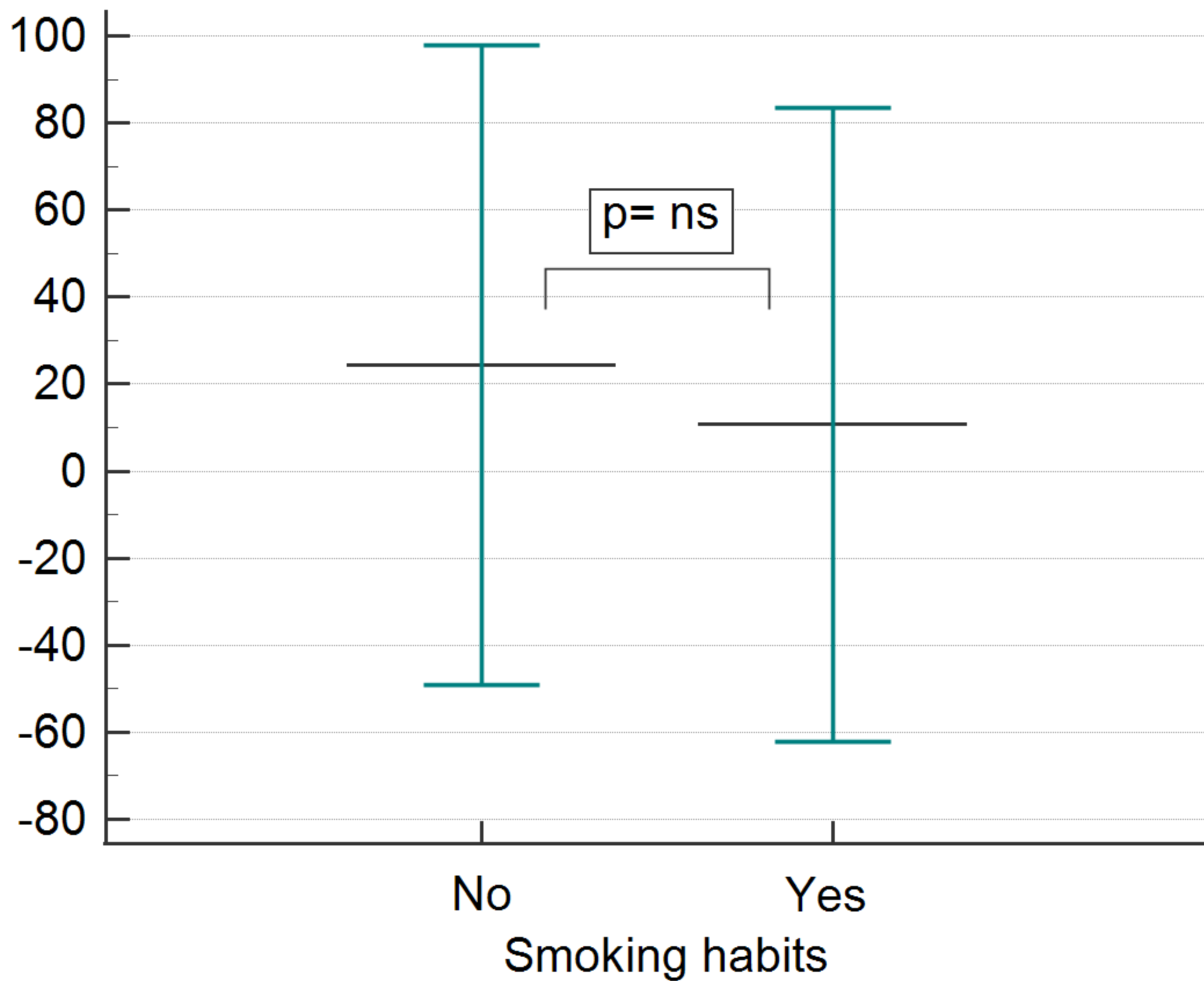

Supplement: Supplementary Figure 2. Association between pre-treatment serum miR-146a expression levels and smoking habits. Ns= not significant [file supplementary_figure_2.pdf]

miR-146a expression levels

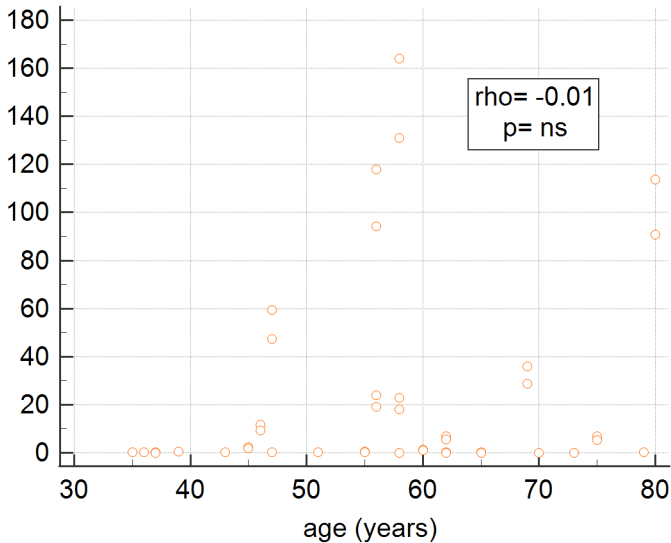

Supplement: Supplementary Figure 3. Correlation between pre-treatment serum miR-146a expression levels and patient’s age at diagnosis. Ns= not significant [file supplementary_figure_3.pdf]

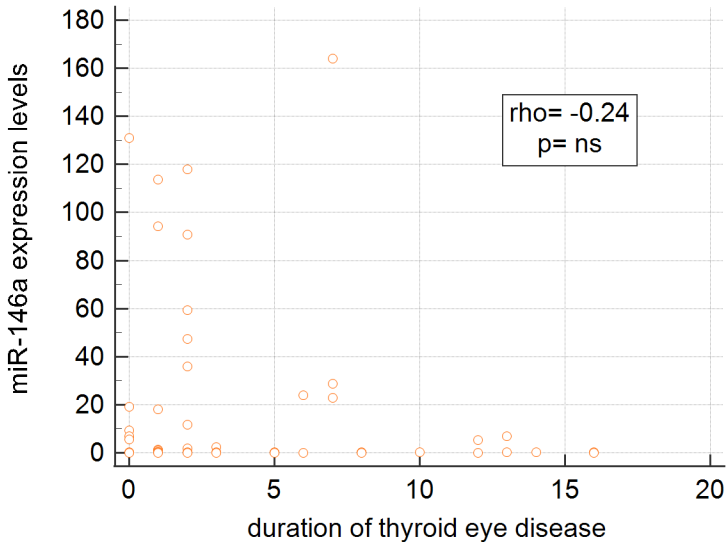

Supplement: Supplementary Figure 4. Correlation between pre-treatment serum miR-146a expression levels and duration of thyroid eye disease. Ns= not significant [file supplementary_figure_4.pdf]
